# Metabolic Pathway of Cardiospecific Troponins: From Fundamental Aspects to Diagnostic Role (Comprehensive Review) 

**DOI:** 10.3389/fmolb.2022.841277

**Published:** 2022-04-19

**Authors:** Aleksey M. Chaulin

**Affiliations:** ^1^ Department of Cardiology and Cardiovascular Surgery, Department of Clinical Chemistry, Samara State Medical University, Samara, Russia; ^2^ Samara Regional Clinical Cardiological Dispensary, Samara, Russia

**Keywords:** cardiospecific troponins, myocardial infarction, metabolic pathway, mechanisms of release of cardiospecific troponins, proteolytic cleavage, elimination of cardiospecific troponins, diagnosis

## Abstract

Many molecules of the human body perform key regulatory functions and are widely used as targets for the development of therapeutic drugs or as specific diagnostic markers. These molecules undergo a significant metabolic pathway, during which they are influenced by a number of factors (biological characteristics, hormones, enzymes, etc.) that can affect molecular metabolism and, as a consequence, the serum concentration or activity of these molecules. Among the most important molecules in the field of cardiology are the molecules of cardiospecific troponins (Tns), which regulate the processes of myocardial contraction/relaxation and are used as markers for the early diagnosis of ischemic necrosis of cardiomyocytes (CMC) in myocardial infarction (MI). The diagnostic value and diagnostic capabilities of cardiospecific Tns have changed significantly after the advent of new (highly sensitive (HS)) detection methods. Thus, early diagnostic algorithms of MI were approved for clinical practice, thanks to which the possibility of rapid diagnosis and determination of optimal tactics for managing patients with MI was opened. Relatively recently, promising directions have also been opened for the use of cardiospecific Tns as prognostic markers both at the early stages of the development of cardiovascular diseases (CVD) (arterial hypertension (AH), heart failure (HF), coronary heart disease (CHD), etc.), and in non-ischemic extra-cardiac pathologies that can negatively affect CMC (for example, sepsis, chronic kidney disease (CKD), chronic obstructive pulmonary disease (COPD), etc.). Recent studies have also shown that cardiospecific Tns are present not only in blood serum, but also in other biological fluids (urine, oral fluid, pericardial fluid, amniotic fluid). Thus, cardiospecific Tns have additional diagnostic capabilities. However, the fundamental aspects of the metabolic pathway of cardiospecific Tns are definitively unknown, in particular, specific mechanisms of release of Tns from CMC in non-ischemic extra-cardiac pathologies, mechanisms of circulation and elimination of Tns from the human body, mechanisms of transport of Tns to other biological fluids and factors that may affect these processes have not been established. In this comprehensive manuscript, all stages of the metabolic pathway are consistently and in detail considered, starting from release from CMC and ending with excretion (removal) from the human body. In addition, the possible diagnostic role of individual stages and mechanisms, influencing factors is analyzed and directions for further research in this area are noted.

## Introduction

Many molecules of our body are widely used as therapeutic targets or as biomarkers for the early and specific diagnosis of various pathologies (Adeva-Andany et al., 2014; Sookoian and Pirola, 2015; Halbrook and Lyssiotis, 2017; Fhu and Ali, 2020). The metabolic pathway of these molecules may affect their concentration or activity, and, consequently, the results of laboratory testing and/or the effectiveness of therapeutic drugs. Therefore, the study of the metabolic pathway and influencing factors is very important for optimizing therapy and laboratory diagnostics.

Notwithstanding the solid achievements in the study of etiopathogenesis, diagnostics, and treatment of acute coronary syndrome (ACS), it still remains one of the leading reasons for disability and mortality of the population in all the developed countries of the world. All patients with ACS are under higher risk of the development of myocardial infarction (MI) and death (Henderson, 2013; Makki et al., 2015; Smith et al., 2015; Chaulin et al., 2020a; Chaulin, 2022a). According to results of the reviews of MI diagnostic criteria conducted in 2012 and 2018 by the European Society of Cardiology (ESC), the American College of Cardiology (ACC), the American Heart Association (AHA) and the World Heart Federation (WHF), the diagnosis verification is based on the presence of myocardial ischemia symptoms (clinical, electrocardiographic, echocardiographic, angiographic) and the positive dynamics of cardiospecific Tn levels in blood of patients (Thygesen et al., 2012; Thygesen et al., 2018; Eckner et al., 2020).

Troponins (Tns) (troponin I (TnI), troponin T (TnT), troponin C (TnC)) are proteins being a part of the troponin complex, which is bound to the protein tropomyosin (Tpm). Tpm, in its turn, together with actin, forms thin filaments of myocytes—the most important component of the contractile apparatus of striated muscle cells (of skeletal and cardiac muscles). All the three Tns participate in calcium-dependent regulation of the striated muscle contraction-relaxation. Each troponin type fulfils specific regulatory functions in contraction-relaxation of striated muscles. TnI is the inhibitory subunit of the Tpm complex that binds actin during relaxation and inhibits the ATPase activity of actomyosin thus preventing muscle contraction in the absence of calcium ions in the cell cytoplasm. TnT is the regulatory subunit, anchoring the troponin complex to thin filaments and, therefore, participating in the calcium-regulated contraction. TnC is the calcium-binding subunit. When the action potential is transferred to the muscle cell, calcium channels in the sarcoplasmic reticulum (“the repository of calcium ions”) open and the sarcoplasmic reticulum releases calcium ions into the sarcoplasm. Then, calcium ions bind to TnC, which leads to the conformational (structural) changes of proteins of the Tn-Tpm complex, as a result of which the Tpm molecule shifts and releases binding sites for the myosin head on the actin filament. It enables the interaction of the myosin head with actin, which underlies the mechanism of contraction of striated muscles (Takeda, 2005; Katrukha, 2013; Henderson et al., 2017; Chaulin, 2021a).

Molecules of Tns have a different amino acid structure depending on their localization in muscles, on the basis of which troponin isoforms are distinguished. Thus, TnI has three isoforms: cardiospecific TnI, TnI of fast-twitch skeletal muscle fibers, and TnI of slow-twitch skeletal muscle fibers. TnT also has three main isoforms: cardiospecific TnT, TnT of fast-twitch skeletal muscle fibers, and TnT of slow-twitch skeletal muscle fibers. According to molecular genetic studies, the amino acid sequence of cardiospecific TnI and cardiospecific TnT differs from the amino acid sequences of the corresponding isoforms of skeletal Tns localized in skeletal muscle fibers by approximately 40–60% (Wei and Jin, 2011; Jin, 2016). This important structural peculiarity allows for the use of cardiospecific TnT and cardiospecific TnI as specific markers for laboratory diagnostics of myocardial injury in MI and other non-cardiac pathological conditions. Cardiospecific TnC, as opposed to TnI and TnT, has completely identical amino acid structure with the muscular (skeletal) TnC, and increased blood levels of this protein will not let us reliably distinguish the cardiac muscle tissue injury from the damage of skeletal muscles, and, therefore, cardiospecific TnС cannot be used as a cardiac marker for MI diagnostics (Wang et al., 2020; Chaulin, 2021b).

**FIGURE 1:**
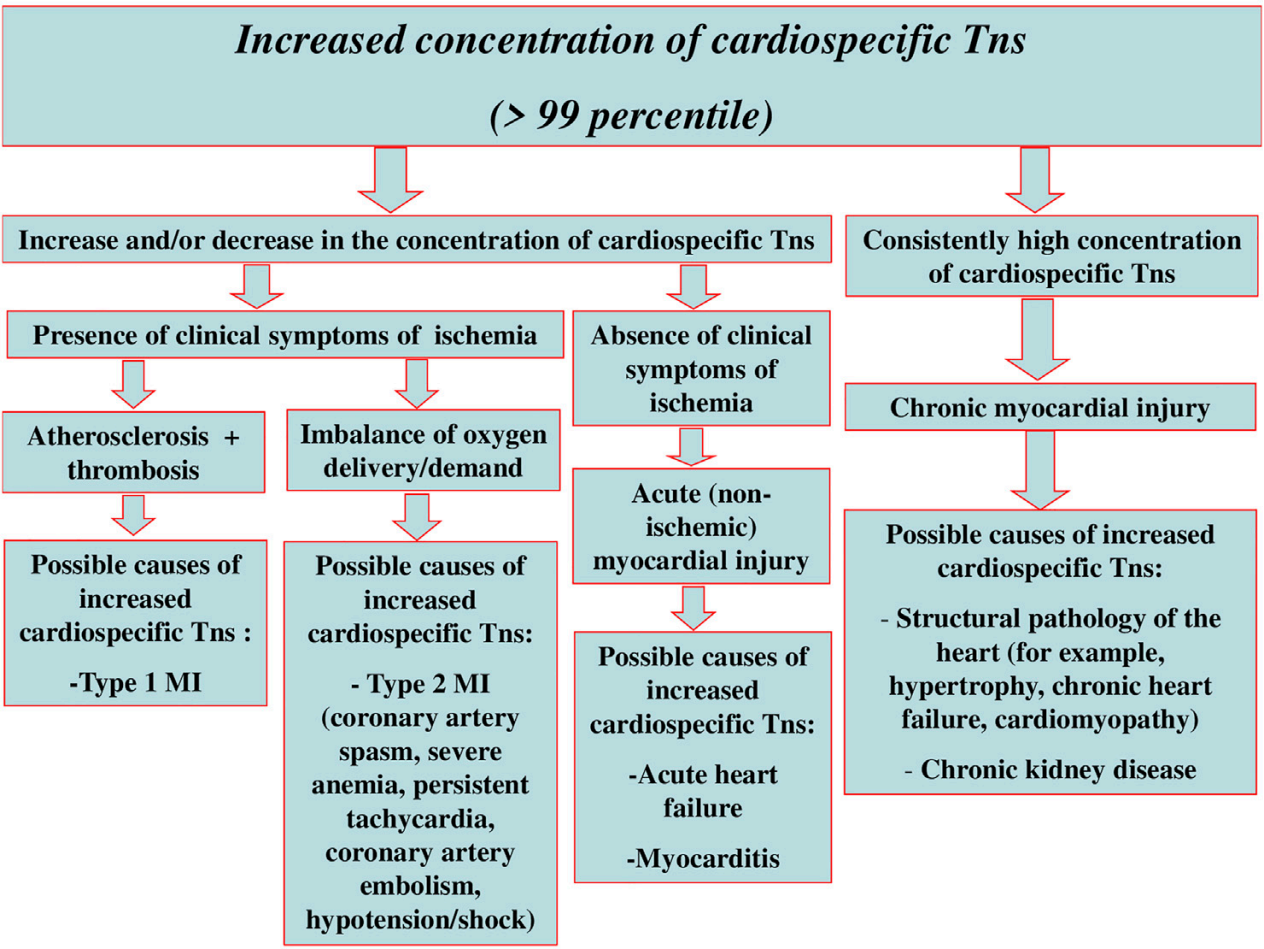
Interpretation of possible reasons for myocardial injury and increase of cardiospecific Tns serum levels.

Although the regulatory documents concerning the diagnostics and treatment of different forms of ACS and MI contain clear recommendations on the time of troponin testing and decision-making levels, and the sensitivity and specificity of most immunoassays approximates 100%, there still remain a number of unsolved problems and issues relating to the application of these markers in clinical practice. Some of these problems are connected with the variety of troponin diagnostic agents, their unequal sensitivity and diagnostic accuracy, different susceptibility to cross-reactive molecules, i.e., with different analytical characteristics of test systems (Apple et al., 2017; Aakre et al., 2020; Chaulin, 2021b). Another range of issues results from the fact that the increase in cardiospecific Tn levels takes place in case of myocardial necrosis of any etiology and sometimes in the absence of irreversible myocardial injury [for instance, in case of reversible injury induced by physical exercises, chronic kidney disease (CKD), or the influence of false-positive factors] (Chaulin and Duplyakov, 2020a; Chaulin and Duplyakov, 2020b; Chaulin et al., 2020b; Chaulin and Duplyakov, 2020c; Chuang et al., 2020; Stavroulakis and George, 2020; Chauin, 2021; Chaulin, 2022b). Besides, along with the necrosis of cardiomyocytes (CMC), there are other mechanisms of cardiospecific Tns release from CMC and/or the increase of cardiospecific Tns concentration in blood serum. Thus, several clinical studies give evidence of very frequent increase in cardiospecific Tns concentration in various pathologies (Lindner et al., 2014; Saad et al., 2015; Wu et al., 2018; Askin et al., 2020; Qin et al., 2021). At the same time, the mechanism of troponin increase in these diseases is not associated with the ischemic necrosis of CMC—the main mechanism of cardiospecific Tn levels increase in MI. The study by G. Lindner et al. is quite representative in this respect. The researchers have conducted the detailed analysis of the reasons (the diseases) causing increase of cardiospecific TnT levels in patients admitted to the emergency department. In total, the study included 1,573 patients, and only 10% of which had the increased level of cardiospecific TnT associated with MI, while all the rest (about 90%) showed no signs of MI, and their increased levels of cardiospecific TnT were induced by other diseases causing the increase of cardiospecific TnT serum levels by non-ischemic mechanisms. The most common reasons of cardiospecific TnT increase were the following ones: pulmonary embolism (PE), CKD, acute aortic dissection, heart failure (HF), acute myocarditis, rhabdomyolysis, application of cardiotoxic chemotherapeutic agents, acute exacerbation of chronic obstructive pulmonary disease (COPD), arterial hypertension (AH), sepsis and infiltrative cardiac pathologies (for example, amyloidosis). The interesting fact revealed by this study was that in 30% of cases the increased levels of cardiospecific TnT were not connected with any previously described causes of cardiospecific Tns increase (Lindner et al., 2014). There is a high probability that these reasons might be connected with the false-positive mechanisms or they have been induced by the factors the researchers and medical practitioners have not paid attention to and have not described yet. Thus, the interpretation of the results showing the increased levels of cardiospecific Tns is an extremely complicated and sometimes even impossible task of modern clinical practice. Therefore, it is important to remember that the troponin test itself is not “the gold standard test” for MI diagnostics, but it can become one only for those patients who show typical clinical symptoms of myocardial ischemia, have corresponding ischemic changes on the electrocardiogram, echocardiogram, etc. Generally, when interpreting possible reasons for the increase of cardiospecific Tns in blood serum, one should be guided by the following schematics (Figure 1).

Due to modern high-sensitive (HS) troponin assays, medical practitioners can carry out early diagnosis of AMI. To do this, it is necessary to evaluate the dynamic changes in serum levels of cardiospecific Tns within 1–2 h after the patient’s admission to the emergency department. The changes (increase) of the concentration of cardiospecific Tn molecules within the first 2 h are very small (may amount to as little as several ng/L) and cannot be detected by moderately sensitive test systems. It should be noted that due to a number of multicenter studies there have been validated algorithms of early diagnostics (0 → 1 h, and 0 → 2 h) of non-ST-segment elevation MI (NSTEMI) for HS test systems of various manufacturers (Table 1) (Collet et al., 2021).

**TABLE 1:** Current diagnostic algorithms for confirmation/exclusion of NSTEMI (0 → 1 h and 0 → 2 h), approved by the ESC (Collet et al., 2021).

					
One-hour NSTEMI diagnostic algorithm										
Troponin immunoassay, company (manufacturer)		Biomarker concentration that indicates an extremely low probability of an NSTEMI diagnosis, ng/L		Biomarker concentration that indicates a low probability of an NSTEMI diagnosis, ng/L		Changes in biomarker concentration after 1 h at which a diagnosis of NSTEMI should be excluded, ng/L		Biomarker concentration that indicates a high probability of an NSTEMI diagnosis, ng/L		Changes in biomarker concentration after 1 h at which a diagnosis of NSTEMI should be confirmed, ng/L
HS-TnT (Elecsys; Roche)		<5		<12		<3		≥52		≥5
HS-TnI (Architect; Abbott)		<4		<5		<2		≥64		≥6
HS-TnI (Centaur; Siemens)		<3		<6		<3		≥120		≥12
HS-TnI (Access; Beckman Coulter)		<4		<5		<4		≥50		≥15
HS-TnI (Clarity; Singulex)		<1		<2		<1		≥30		≥6
HS-TnI (Vitros; Clinical Diagnostics)		<1		<2		<1		≥40		≥4
HS-TnI (Pathfast; LSI Medience)		<3		<4		<3		≥90		≥20
Troponin immunoassay, company (manufacturer)		Biomarker concentration that indicates an extremely low probability of an NSTEMI diagnosis, ng/L		Biomarker concentration that indicates a low probability of an NSTEMI diagnosis, ng/L		Changes in biomarker concentration after 2 h at which a diagnosis of NSTEMI should be excluded, ng/L		Biomarker concentration that indicates a high probability of an NSTEMI diagnosis, ng/L		Changes in biomarker concentration after 2 h at which a diagnosis of NSTEMI should be confirmed, ng/L
HS-TnT (Elecsys; Roche)		<5		<14		<4		≥52		≥10
HS-TnI (Architect; Abbott)		<4		<6		<2		≥64		≥15
HS-TnI (Centaur; Siemens)		<3		<8		<7		≥120		≥20
HS-TnI (Access; Beckman Coulter)		<4		<5		<5		≥50		≥20
HS-TnI (Clarity; Singulex)		<1		to be determined		to be determined		≥30		to be determined
HS-TnI (Vitros; Clinical Diagnostics)		<1		to be determined		to be determined		≥40		to be determined
HS-TnI (Pathfast; LSI Medience)		<3		to be determined		to be determined		≥90		to be determined

According to the data of modern (HS) troponin tests, cardiospecific Tn molecules are detected in blood and a number of other biological fluids in all healthy people (Koerbin et al., 2012; Mirzaii-Dizgah and Riahi, 2013a; Abe et al., 2018; Garcia-OsunaMirzaii-Dizgah and Riahi, 2013a; Abe et al., 2018; Garcia-Osuna et al., 2018; Chaulin et al., 2019; Bahadur et al., 2020; Chaulin et al., 2020c; Chen et al., 2020; Odsæter et al., 2020). This circumstance poses new questions and issues for researchers to find and explain possible mechanisms underlying the release of troponin molecules from intact myocardial cells, in particular, the need to find and justify possible release mechanisms of cardiospecific troponin molecules from intact CMC. Hence, the molecules of cardiospecific Tns can be considered normal products of cardiac muscle tissue metabolism. However, the precise mechanisms of the release are not clear yet and are hypothetical. Moreover, the factors that may influence and facilitate or, on the contrary, reduce the release of troponins will be of great importance for researchers and medical practitioners. Currently, the most discussable biological factors influencing the degree of troponin release from healthy myocardium are the gender, age, and circadian characteristics (Gore et al., 2014; Klinkenberg et al., 2014; Fournier et al., 2017; Boeddinghaus et al., 2018; Monneret et al., 2018; Bohn et al., 2019; Rocco et al., 2019; Romiti et al., 2019; Giannitsis et al., 2020; Chaulin and Duplyakov, 2021a). Gender-related characteristics of cardiospecific Tns consist in the fact that the myocardium of healthy men releases more molecules of cardiospecific Tns than that of healthy women. These characteristics are validated by many clinical studies, and practically in all modern test systems, it is recommended to use the threshold values (99th percentile) in accordance with gender (Eggers and Lindahl, 2017; Rocco et al., 2019; Chaulin et al., 2021a). The age-related characteristics of cardiospecific Tns are that a greater amount of cardiospecific Tn molecules are released from the myocardium of elderly patients compared to the myocardium of young people (Gore et al., 2014; Hickman et al., 2019; Sedighi et al., 2019). The circadian features of cardiospecific Tns are that more cardiac troponin molecules are released from the myocardium of healthy people in the morning than from the myocardium of healthy people in the evening-night period (Chaulin et al., 2020d; Chaulin and Duplyakov, 2021b). It should be noted that the age and circadian characteristics of cardiac troponins are not typical for all test systems, and according to some studies, they are contradictory (Vogiatzis, 2018; Wildi et al., 2018; Zaninotto et al., 2020). Before using these characteristics in rapid diagnostic algorithms, it is necessary to conduct additional large studies to validate the age and circadian characteristics of cardiospecific Tns.

One of the significant problems of both moderately sensitive and modern highly sensitive (HS) immunoassays is the lack of their standardization (Tate et al., 2010; Panteghini et al., 2008; Jarolim, 2015). This leads to the fact that different troponin immunoassays detect different values (concentrations) of cardiospecific Tn molecules in blood and other biological fluids of the same patient. So, in accordance with the data in Table 1, the threshold levels for excluding/confirming NSTEMI differ by several times when using immunoassays from different manufacturers (Collet et al., 2021). Based on this, we can say that each method detects, in fact, different molecules of cardiospecific Tns and their fragments in a biological fluid. This creates certain difficulties and problems: 1) the need to validate the threshold concentrations of cardiospecific Tns for each test system, including newly developed ones, which is associated with additional high costs; 2) the need for a thorough study of interfering factors for each of the known detection methods that also involves additional costs; 3) upon admission of a patient to the hospital, dynamic changes in cardiospecific Tns to confirm/exclude MI during the first and subsequent hours can be traced and evaluated only when using the same test system, and immunoassays from different manufacturers cannot be used for this goal. At the same time, different institutions can use different test systems, which will not allow for proper assessment of the dynamic changes in case of urgently required transportation of a patient to another institution, which will be associated with a loss of time for additional examinations and additional economic costs.

It should be noted separately that the molecules of cardiospecific Tns can be affected by very numerous proteolytic enzymes present in blood, which thereby can indirectly affect the levels of troponins in blood of patients. For example, due to an increase in the activity of proteases that cause fragmentation of troponin molecules, the duration of circulation (half-life) of troponins in the bloodstream will decrease, which can potentially lead to false-negative results when using those test systems that have diagnostic antibodies directed against fragmented epitopes of troponin molecules (Streng et al., 2013; Katrukha et al., 2018). A decrease in the activity of such proteolytic enzymes, on the contrary, may hypothetically lead to false-positive results. However, the current specific knowledge about this metabolic stage, in particular, the exact information about all the influencing enzymes and mechanisms of troponin fragmentation, is extremely scarce and is not taken into account in clinical practice. This stage of troponin metabolism and its effect on diagnostics will be considered in more detail in this manuscript below in the paragraph on the circulation of cardiospecific Tns in blood plasma.

A very interesting direction in studying the diagnostic value of cardiospecific Tns is the assessment of the possibility of using other biological fluids as biomaterials for detection of troponin molecules. This direction is developing due to an increase in the sensitivity of immunoassays [the creation of highly sensitive (HS) test systems], which can detect very low concentrations (at levels of several ng/L) of troponins that circulate in many biological fluids, including non-invasively obtained fluids (urine and oral fluid) (Mirzaii-Dizgah and Riahi, 2013a; Mirzaii-Dizgah and Riahi, 2013b; Klichowska-Palonka et al., 2015; Abdul Rehman et al., 2017; Pervan et al., 2017; Garcia-Osuna et al., 2018; Mishra et al., 2018; Chaulin et al., 2019; Chaulin et al., 2020c; Chen et al., 2020; Bahbah et al., 2021; Chaulin, 2021c). Moderately sensitive methods, as a rule, cannot detect such low concentrations of cardiospecific Tn molecules present in these biological fluids (Ellis et al., 2001; Ziebig et al., 2003). The mechanisms of penetration/transport of cardiospecific Tns into these biological fluids should also be considered as one of the stages of the metabolic pathway of cardiospecific Tns. And the study and understanding of precise mechanisms of penetration/transport of cardiospecific Tns will increase their diagnostic value and validate new methods for diagnosing cardiovascular diseases (CVD) through the use of other biological fluids, in particular non-invasively obtained fluids, since their collection has a number of advantages (for example, painlessness and atraumatic nature, lower risk of introduction of blood-borne infections and the possibility of obtaining biomaterial without the involvement of medical personnel) over the use of blood as a biomaterial. In addition, there are prospects for the creation of specialized diagnostic test strips (“dry chemistry” methods) for the detection of troponins in urine and/or oral fluid, which will make it possible to carry out express diagnostics and/or monitoring of CVD at home by patients themselves or by their relatives.

The determination of cardiospecific Tns in pericardial fluid can be used in forensic medicine to determine the cause of death. Cardiomarkers, including cardiospecific Tns, are able to passively diffuse into the pericardial fluid, being found in higher levels in this fluid than in blood serum (Batalis et al., 2010; Palmiere et al., 2018; Hernández-Romero et al., 2021). So in a recent study D. Hernández-Romero et al. showed that the levels of cardiospecific TnI in pericardial fluid and the coefficient of cardiospecific TnI (the ratio of TnI levels in pericardial fluid and blood serum) are associated with the cause of sudden death. The highest values of the cTnI coefficient are noted at death from MI, and lower values of the cTnI coefficient are noted at death from asphyxia, injuries and other natural causes (Hernández-Romero et al., 2021).

The main biological fluids in which the molecules of cardiospecific Tns are detected and their diagnostic value are summarized in Table 2.

**TABLE 2:** Biological fluids in which the molecules of cardiospecific Tns are detected and the diagnostic role.

Biological fluid	Diagnostic role of cardiospecific Tns	Sources
Blood (whole, serum, plasma)	It is the main biological fluid used to diagnose AMI and assess the prognosis of patients suffering from non-ischemic cardiac (myocardites, Takotsubo syndrome, cardiomyopathies, etc.) and non-cardiac (sepsis, renal failure, neurogenic pathologies, etc.) pathologies that cause damage to CMC.	Lindner et al. (2014), Wu et al. (2018), Chaulin and Duplyakov (2020b), Chaulin and Duplyakov (2020c), Chuang et al. (2020), Stavroulakis and George (2020); Chauin (2021), Chaulin (2022b)
Urine	Molecules of cardiospecific Tns can be detected in this biological fluid via highly sensitive test systems. Increased troponin levels have a high prognostic value in diabetes mellitus and AH. The method of obtaining this biological fluid is non-invasive, which has a number of advantages over the use of blood. It should be noted that the possibilities of examination of HS troponins in urine are still poorly studied and have not been finally validated. Further research is needed before the introduction of this method into clinical practice	Mirzaii-Dizgah and Riahi (2013b), Odsæter et al. (2020)
Oral fluid	The levels of cardiospecific Tns in oral fluid increase in AMI and moderately correlate with serum troponin levels; therefore, further study of this area of non-invasive diagnostics is very promising	Panteghini et al. (2008), Koerbin et al. (2012), Abe et al. (2018), Bahadur et al. (2020), Bahbah et al. (2021)
Pericardial fluid and cerebrospinal fluid	Molecules of cardiospecific Tns are detected in pericardial fluid and cerebrospinal fluid via moderately sensitive and HS test systems and, according to some studies, may correlate with serum levels of cardiospecific Tns. Increased cardiospecific Tn levels in these biological fluids may reflect the degree of myocardial damage and may be used in forensic medicine to determine the cause of death. However, due to the relative paucity of such studies, further investigation of these possibilities is necessary	Ellis et al. (2001), Ziebig et al. (2003), Mishra et al. (2018), Palmiere et al. (2018), Chaulin (2021c); Hernández-Romero et al. (2021)
Amniotic fluid	Cardiospecific Tn molecules can be detected in amniotic fluid via moderately sensitive and HS-Tn immunoassays. Increased cardiospecific troponin levels may indicate chronic fetal hypoxia, abnormal development of the cardiovascular system and fetal myocardial injury, and an increased risk of fetal death during the intrauterine growth period. However, it is worth noting that such studies are few in number. Further research is needed to clarify the diagnostic capabilities of amniotic fluid	Maeda et al. (2009), Wang et al. (2011), Chen et al. (2015), González-Herrera et al. (2016)

## Metabolic Pathway of Cardiospecific Tns

**FIGURE 2:**
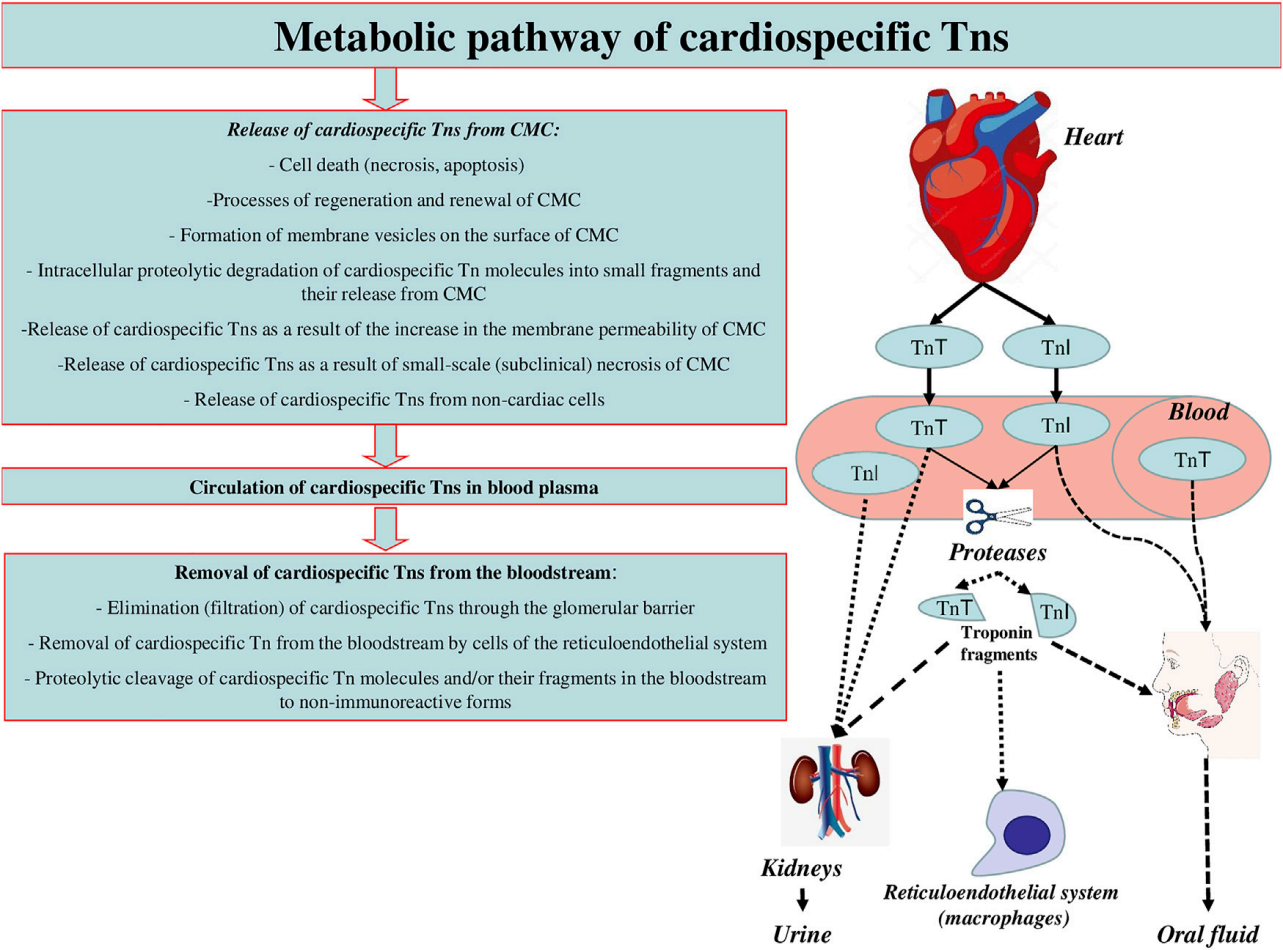
Metabolic pathway of cardiospecific Tns.

Conventionally, there can be distinguished three main stages of the metabolic pathway of cardiospecific Tns (Figure 2):

► Release of cardiospecific Tns from CMC
► Circulation of c cardiospecific Tns in blood plasma
► Removal (elimination) of cardiospecific Tns from the bloodstream. Similar key stages of the metabolism of molecules are distinguished for other molecules in order to conveniently and consistently consider the main metabolic characteristics of molecules.

Each of these stages of metabolism can play a decisive role in the regulation of the concentrations of cardiospecific Tns in blood, i.e., their diagnostic value. In addition, there are a number of factors that can have a potential and hypothetical influence on these stages of the metabolic pathway of cardiospecific Tns. These factors can be physiological conditions, for example, gender, age and circadian characteristics, which have a certain effect on the degree of release of troponin molecules from the myocardium of healthy people. Or, changes in the activity of proteolytic enzymes that target cardiospecific Tns molecules. The activity of proteolytic enzymes can also change under pathological conditions and/or in case of taking certain medications. CKD can be noted as an example of a significant factor affecting the removal of cardiospecific Tns from the bloodstream. It is important to emphasize that there may be an extremely large number of such factors, and some of them are probably still unknown.

Below I will sequentially consider each of the stages of the metabolic pathway of cardiospecific Tn and note the known and assumed factors that may affect these mechanisms and the diagnostic role of cardiospecific Tns.

## Release of Cardiospecific Tns From CMC: Mechanisms and Diagnostic Role

The introduction of HS test systems into practice made it possible with high accuracy (variability of the analysis within 10%) to detect very low concentrations of cardiospecific Tns, ranging from 0.001 to 0.01 ng/ml and below the values corresponding to the 99th percentile (upper limit of the norm). As a result, cardiospecific Tn molecules were found in almost 100% of healthy people, and instead of a clear borderline level typical of AMI, a smooth scale appeared, capable of reflecting subclinical myocardial pathology associated with structural (non-ischemic) damage, stable coronary artery diseases and other pathological conditions that negatively affect CMC (Lindner et al., 2014; Ji et al., 2016; Kavsak, 2018; Wu et al., 2018; Chaulin et al., 2021b; Chaulin and Duplyakov, 2021c; Chauin, 2021; Chaulin, 2022b). Considering the fact that the molecules of cardiospecific Tns began to be detected in all healthy people, it became necessary to study and explain the mechanisms of the release of cardiospecific Tns from the intact myocardium. In this regard, the researchers are discussing the following possible mechanisms for the release of cardiospecific Tn molecules and an increase in their serum levels: a) the release of cardiospecific Tns as a result of the processes of regeneration and renewal of CMC, b) the release of cardiospecific Tns as a result of apoptosis of CMC, c) the release of cardiospecific Tns as a result of formation of membrane vesicles on the surface of CMC, d) intracellular proteolytic degradation of cardiospecific Tn molecules into small fragments and the release of the latter through the intact membrane of CMC, e) release of cardiospecific Tns as a result of the increase in the membrane permeability of CMC, f) release of cardiospecific Tns as a result of small-scale (subclinical) necrosis of CMC, g) the release of cardiospecific Tns from non-cardiac cells. Some of the above mechanisms of release can not only explain the detectable concentrations of cardiospecific Tns in healthy individuals, but also significantly activate/increase under certain physiological conditions and pathological processes (Jaffe and Wu, 2012; Hammarsten et al., 2018; Mair et al., 2018; Tjora et al., 2018). For example, apoptosis of CMC can increase with an increase in blood pressure (Gumprecht et al., 2019; Chaulin and Duplyakov, 2021d), stretching of the myocardial walls (Liao et al., 2004; Cheng et al., 2015), increased stimulation of beta-adrenergic receptors (Communal and Colucci, 2005; Dalal et al., 2012; Jiang et al., 2017) and a number of other mechanisms (Chen and Tu, 2002; Kunapuli et al., 2006), which thereby may facilitate the release of cardiospecific Tns from CMC. And in conditions of CKD, the expression of cardiospecific Tns in skeletal muscles is noted (Ricchiuti and Apple, 1999), which, according to some authors, can lead to an increase in serum concentrations of cardiospecific Tns in patients with CKD (Ricchiuti et al., 1998; Ricchiuti and Apple, 1999).

Below, I will consider each of the above mechanisms of cardiospecific Tns release sequentially and in more detail.

## Release of Cardiospecific Tns as a Result of the Processes of Regeneration and Renewal of CMC

Due to studying the metabolism of C^14^-labeled DNA molecules in CMC, the researchers proved the presence of regeneration and renewal of CMC. There was carried out a long-term observation of people with the inclusion of a radioactive isotope of carbon (C^14^) in the DNA of CMC, which occurred as a result of nuclear weapons tests. The authors calculated the rate of renewal of CMC by studying the rate of DNA synthesis, which was calculated by investigating the rate of accumulation of C^14^ in CMC. There was found the renewal of CMC, the intensity of which decreased annually—from 1% per year at the age of 25 years to 0.45% per year at the age of 75 years. In general, about 50% of CMC underwent renewal throughout life (Bergmann et al., 2009; Bergmann et al., 2012). These results indicate the existence of a small regenerative potential in CMC. Some researchers suggest that the process of renewal of CMC may be associated with the release of cardiospecific Tns from CMC (White, 2011), however, the specific mechanism underlying this phenomenon remains unknown. As a possible hypothesis, it can be assumed that intracellular molecules of cardiac markers, including cardiospecific Tns, will be released from gradually aging and naturally dying CMC as a result of the gradual destruction of the cell membrane. Since the rate of renewal of CMC is low, the degree of increase in serum levels of cardiospecific Tns is also insignificant (no higher than the 99th percentile). Thus, this mechanism can hypothetically explain the presence of a small amount of cardiospecific Tn molecules in the bloodstream of all healthy individuals.

In accordance with the data of other researchers, the average rate of CMC renewal in mammals is 0.5–2.0% per year and can vary depending on the influence of certain factors, such as physiological conditions (physical activity), trauma and concomitant diseases (Foglia and Poss, 2016; Lázár et al., 2017; Nakada et al., 2017). Thus, the rate of renewal of CMC increases significantly after myocardial damage. An experimental study conducted by P. Docshin et al. has shown that ischemic myocardial injury causes the activation of endogenous stem cells and increases the rate of CMC renewal (Docshin et al., 2018). Two other research groups led by C. Waring et al. (Waring et al., 2014) and M. Rovira et al. (Rovira et al., 2018; Schüttler et al., 2019) revealed an increase in the processes of proliferation and differentiation of stem cells in the myocardium of rats and zebrafish (*Danio rerio*).

However, the assessment of the degree of regeneration and the rate of renewal of CMC can be significantly influenced by inflammatory processes, proliferation of non-myocyte cells and the formation of a connective tissue scar in the myocardium, which often complicate and/or distort investigation results (Talman and Ruskoaho, 2016; Isomi et al., 2019). Further research is needed to investigate the specific role of CMC regeneration and renewal in the release of cardiospecific Tns from CMC.

## Release of Cardiospecific Tns as a Result of Apoptosis of CMC

To date, a large number of factors have been discovered that can trigger the processes of apoptosis of CMC (Lee and Gustafsson, 2009; Zhang et al., 2019). Induction of apoptosis leads to an increase in the activity of caspases (proteolytic enzymes of the cysteine protease family), which can fragment (damage) DNA and protein molecules, leading to cell death. In contrast to necrosis, during apoptosis, the cell dies more slowly, the integrity of the cell membrane remains much longer, and the inflammatory reaction around the dead cell is not observed. To study the processes of apoptosis, many methods are used: various types of microscopy (light, electron, and fluorescence), flow cytometry, immunohistochemical analysis, the TUNEL method [Terminal deoxynucleotidyl transferase (TdT) dUTP Nick-End Labeling], etc. The TUNEL method is the most reliable and early method for detecting apoptosis. This method allows visualization of cell nuclei in which the DNA molecule has been fragmented due to increased activity of endonucleases and caspases. This method is most often used in all modern studies aimed at the exploration of the etiopathogenetic mechanisms of apoptosis of various cells, including CMC (Zorc-Pleskovic et al., 2006; Kyrylkova et al., 2012; Zhang et al., 2018).

An experimental study led by B. Weil et al. has shown that short-term ischemia activates apoptosis of CMC in experimental animals, and apoptosis of CMC is accompanied by an increase in serum levels of cardiospecific Tns. Short-term myocardial ischemia was simulated by means of balloon occlusion of a branch of the left coronary artery and the fact of occlusion was confirmed by coronary angiography. The duration of ischemia was 10 min, after which reperfusion was carried out by deflation of the balloon. To confirm the apoptosis of CMC, the TUNEL method was used, according to the results of which the number of CMC in the state of apoptosis was significantly increased (6 times compared with the control group of animals). At the same time, no histological signs of myocardial necrosis were observed. This suggests that short-term (in this case, 10-min) ischemia does not cause ischemic necrosis of CMC, but enhances apoptotic processes in the myocardium. However, the levels of cardiospecific Tns began to rise rapidly: 30 min after reperfusion, the cardiospecific TnI concentration approached the upper limit of the norm (38 ng/L) and after 1 h exceeded it (51 ± 17 ng/L). Two and 3 h after reperfusion, the serum levels of cardiospecific TnI were 148 ± 88 and 180 ± 117 ng/L, respectively, which indicated the continuing release of troponin molecules from the myocardium. And, finally, 24 h after reperfusion, the cardiospecific TnI concentration reached its peak and amounted to 1,021 ± 574 ng/L (Weil et al., 2017). Thus, this experimental study elegantly demonstrates the role of apoptosis (induced by short-term ischemia) in the release of cardiospecific Tn molecules from CMC. The limitation of this study is the relatively short interval of investigating the cardiac muscle tissue for the presence of histopathological changes; according to these results, it is impossible to determine the degree and reversibility of damage to CMC during apoptosis induced by short-term ischemia. Besides, for detection of cardiospecific TnI, there was used a moderately sensitive test system, which is inferior in diagnostic capabilities to modern (HS) immunoassays. In this regard, the dynamic changes in the levels of cardiospecific Tns detected by HS immunoassays during apoptosis could be significantly different, especially in the first minutes and hours after reperfusion.

The literature also describes other situations when apoptosis of CMC is induced by other mechanisms that are not associated with reversible (short-term) ischemia of cardiac muscle tissue. The authors identify the following mechanisms of apoptosis that can promote the release of cardiospecific Tns from CMC: stretching of the myocardial walls, increased preload on the heart, and increased activity of the sympathoadrenal system (Cheng et al., 1995; Communal and Colucci, 2005; Jiang et al., 2017). Thus, W. Cheng et al. reported that apoptosis of CMC increases with stretching of the myocardial walls (Cheng et al., 1995). This allows us to consider many physiological and pathological conditions that cause stretching of the myocardial walls as possible inducers of apoptosis and thus can, to some extent, explain the increased serum levels in patients after prolonged and intense physical exertion or having AH, PE, COPD and a number of other pathologies (Cheng et al., 1995; Park et al., 2017; Gherasim, 2019; Aengevaeren et al., 2021).

In another experimental study, B. Weil et al. concluded that an increase in preload on the heart triggers apoptosis and causes an increase in the concentration of cardiospecific TnI in blood of experimental animals. To increase the preload on the heart, the experimental group of animals received intravenous drug—phenylephrine (300 μg of the drug per minute) for 1 h. After the simulation, echocardiography was used to confirm myocardial overload, and to verify apoptosis and determine cardiospecific TnI levels, there were used histological methods, including the TUNEL method and moderately sensitive immunoassay, respectively. As a result of histological examination of the myocardium of the experimental group of animals, there was noted a significant increase in the number of CMC in the state of apoptosis, as compared to the control group. At the same time, no histological signs of myocardial necrosis were recorded. 24 h after the simulation, the number of CMC in the state of apoptosis decreased to the level of the control group, which indicates the reversibility of apoptotic changes. The cardiospecific TnI concentration exceeded the upper limit of the norm 30 min after the simulation, and then the cardiospecific TnI levels continued to rise sharply and reached a value of 856 ± 956 ng/L 1 h after the simulation. Serum levels of cardiospecific TnI remained elevated throughout the study period (24 h) and peaked at 1,462 ± 1,691 ng/L (Weil et al., 2018). Since signs of necrosis, unlike apoptosis, were not observed, it should be considered that apoptosis induced by myocardial overload plays an important role in the release of cardiospecific TnI molecules from CMC cells (Felker and Fudim, 2018).

Injection of a sympathetic stimulator also increases afterload, which ultimately increases both systemic vascular resistance (SVR) and end-diastolic pressure (EDP) (Felker and Fudim, 2018; Singam et al., 2020). Thus, myocardial afterload also causes pathological remodeling of the myocardium and ultimately hypertrophy, leading to heart failure, apoptosis of CMC and the release of cardiospecific Tns into the bloodstream.

The degree of release of cardiospecific Tn molecules from CMC as a result of apoptosis induced by myocardial overload depends on the strength and duration of exposure. For example, relatively small myocardial overload is observed on mild to moderate exertion, in AH and non-massive PE, so the increase in serum levels of cardiospecific Tns in these conditions is also relatively small. But, for example, on high-intensity exertion or in massive PE, myocardial overload becomes much more significant, therefore, these conditions are accompanied by relatively higher serum levels of cardiospecific Tns (Sanchez et al., 2008; Daquarti et al., 2016; El-Menyar et al., 2019).

Another very interesting mechanism for the initiation of apoptosis is an increase in the activity of the sympathoadrenal system. A research group led by K. Singh et al. found that stimulation of beta-adrenergic receptors (β-AR) regulates intracellular apoptotic signaling pathways in CMC. Moreover, stimulation of β1-AR enhances apoptosis of CMC, while stimulation of β2-AR has the opposite effect (Colucci et al., 2000; Singh et al., 2000). It was also noted that the density of β-AR subtypes changes significantly with age (Xiao et al., 1998). Thus, in elderly patients, a more pronounced decrease in the number of β2-AR is noted, which may contribute to a weakening of the anti-apoptotic effect and, accordingly, an increase in apoptosis of CMC (Xiao et al., 1998; Lucia et al., 2018; Mougenot et al., 2019). A higher degree of apoptosis in elderly patients can probably be associated with age-related characteristics of cardiospecific Tn levels: in older people, cardiospecific Tn levels are significantly higher than in young people. The age-related characteristics of cardiospecific Tns have been demonstrated in a number of clinical studies through blood examination with HS test systems (Gore et al., 2014; Boeddinghaus et al., 2018; Monneret et al., 2018; Bohn et al., 2019; Romiti et al., 2019).

Considering the above, apoptosis of CMC should be considered as a significant mechanism for the release of cardiospecific Tn molecules from CMC. This mechanism is not associated with necrosis of CMC and contributes to a very significant increase in serum levels of cardiospecific Tns. Thus, apoptosis of CMC can be of great diagnostic value in conditions such as prolonged and intense physical activity, AH, PE, HF, and, probably, in old age. Further research is needed to clarify the exact role of apoptosis in the release of cardiospecific Tns in physiological and pathological conditions.

## Release of Cardiospecific Tns as a Result of the Formation of Membrane Vesicles on the Surface of CMC

This mechanism of release of cardiospecific Tns from CMC was first described in an experimental study relatively long ago. A research group led by P. Schwartz reported that on the plasma membrane of CMC membrane vesicles (blebbing vesicles) are formed (Schwartz et al., 1984; Siegmund et al., 1990). And during ischemia, the number of blebbing vesicles increases in comparison with intact CMC. A similar trend is also typical for hepatocytes (Piper et al., 1984). Since these vesicles are formed from fragments of the cell membrane and the cytoplasm of CMC, these vesicles may contain some cytoplasmic proteins of CMC, in particular cardiac markers (creatine kinase MB isoform, myoglobin and the cytoplasmic fraction of cardiospecific Tns, and others). However, since the volume of the cytoplasmic fraction of cardiospecific Tns is small (approximately 3–4% for cardiospecific TnI and 7–8% for cardiospecific TnT of the total amount of cardiospecific troponins in the CMC) (Chaulin, 2021d), the contribution of this mechanism to the degree of increase in serum levels of cardiospecific troponins will also be limited. Based on the peculiarities of the formation of blebbing vesicles (a significant increase in ischemia), it can be assumed that this mechanism is involved in the release of cardiospecific Tns in those pathological conditions that are accompanied by ischemia of CMC at an early stage. For example, the initial (prenecrotic) stage of myocardial ischemia can provoke the formation of blebbing vesicles and the release of troponins into the bloodstream, which will lead to the formation of the first peak in serum concentrations of cardiospecific Tns. Subsequently, two main scenarios are possible: 1) with a decrease of ischemia of CMC, the formation of blebbing vesicles stops and cardiospecific Tn concentrations quickly return to normal, 2) with the continuation/intensification of ischemia (as, for example, during MI), the formation of blebbing vesicles increases, and, in addition to this, there occur the destruction of the plasma membrane of CMC and proteolysis (fragmentation) of cardiospecific Tn proteins, which are part of the main (structural or contractile) troponin fraction, which will lead to the formation of the second peak in serum concentrations of cardiospecific Tns. From a pathogenetic point of view, any physiological or pathological condition that will lead to ischemia of CMC (even reversible myocardial ischemia) can activate this mechanism of cardiospecific Tn release. For instance, some physiological conditions (physical activity) (Aakre and Omland, 2019) or pathological conditions (sepsis) (Sheyin et al., 2015) can cause an increase in the oxygen demand of CMC, which, accordingly, will be accompanied by ischemia of the cardiac muscle tissue.

## Intracellular Proteolytic Degradation of Cardiospecific Tn Molecules Into Small Fragments and Their Release From CMC

The size and location of intracellular molecules are two key factors that affect the transport (release) of molecules across the cell membrane. Low molecular weight biomarkers are much more intensively released across the plasma membrane, which plays a role in the diagnostics of many diseases, including CVD. So, for example, with the development of MI, the concentration of low molecular weight cardiac markers (myoglobin) in blood serum rises much earlier than the concentration of high molecular weight cardiac markers (lactate dehydrogenase-1) (Brian Gibler et al., 1987; Bhayana and Henderson, 1995; Chen et al., 2019). This is due to the fact that myoglobin molecules are small and can be released at the initial stages of ischemia during the development of MI (when the plasma membrane of CMC is still relatively insignificantly damaged). A larger molecule (lactate dehydrogenase-1) can leave the CMC only when its cell membrane is significantly damaged. Biomarkers that are freely localized in the cytoplasm of cells (for example, myoglobin, cytoplasmic (non-contractile) fraction of troponins) also have advantages when released from the cell, in contrast to those biomarkers that are localized in organelles (nucleus or mitochondria of cells) or are tightly bound to structural components of sarcoplasm (for example, the structural fraction of cardiospecific Tns involved in the regulation of the contractile fraction of the myocardium). So, during the development of MI, the primarily released molecules are cardiospecific Tn molecules that are part of the cytoplasmic fraction of cardiospecific Tns, and only then there takes place the destruction of sarcomeres, in particular of the Tn-Tpm complex, and the release of structural cardiospecific Tns.

The most important factor that can affect the size of a molecule (biomarker) and, accordingly, the possibility of its release, is the degree of activity of enzymes that cause proteolysis (fragmentation) of this molecule (McDonough et al., 1999; Feng et al., 2001). The activity of proteolytic enzymes can change both under physiological and pathological conditions. In an experimental study conducted by J. Feng et al., it was demonstrated that an increase in preload on cardiac muscle tissue activates the enzyme calpain, which fragments the cardiospecific TnI molecule, which could potentially play a role in the release of this biomarker from CMC and an increase in its level in blood serum (Feng et al., 2001). Thus, physiological and pathological conditions causing an increase in the preload on the myocardial wall can promote the release of cardiospecific Tns from CMC by this mechanism.

In addition to the enzyme calpain, the cleavage of cardiospecific Tn molecules can be catalyzed by some types of matrix metalloproteinases (MMP 2 and MMP 14) (Feng et al., 2001; Qun Gao et al., 2003; Lin et al., 2013; Parente et al., 2021) and the enzyme thrombin (Streng et al., 2016; Bodor, 2017; Katrukha et al., 2017). The activity of these enzymes can also be influenced by pathological processes and some drugs, which thereby hypothetically can affect the serum levels of cardiospecific Tns. For example, an increase of thrombin activity in patients with dilated cardiomyopathy (Ito et al., 2013; Ito et al., 2017) hypothetically can contribute to the fragmentation of cardiospecific TnT, which can have both pathogenetic significance (damage to cardiospecific TnT, which is one of the main components of the contractile apparatus of CMC), and diagnostic value: a decrease in the size of the cardiospecific TnT molecule as a result of fragmentation and a possible increase in the release of these fragments into the bloodstream. However, for the final verification of this mechanism (thrombin-induced intracellular cleavage of cardiospecific troponin T) and its role in the pathogenesis of cardiomyopathies, an experimental study must be performed.

Changes in acidity (pH) can also modulate the activity of intracellular proteolytic enzymes (Matsukura et al., 1981; Peng et al., 2021). So, pathological conditions that disrupt myocardial metabolism, in particular myocardial ischemia, lead to a switch from aerobic myocardial metabolism to anaerobic metabolism and an increase in the formation of lactic acid, which will shift the pH towards acidosis. Under conditions of acidosis, then, proteolytic and proapoptotic enzymes will be activated (Feng et al., 2001; Hickman et al., 2010; Peng et al., 2021), which, through fragmentation, will promote the formation of many small fragments (molecules) of cardiospecific Tns, which will increase the likelihood of their release from CMC into the bloodstream.

## Release of Cardiospecific Tns as a Result of Increased Membrane Permeability of CMC

The membrane permeability of CMC is an important factor that plays a role in the release of cardiac marker molecules from CMC into the bloodstream (Chaulin, 2021e). Based on the analysis of the results of existing experimental data, two main mechanisms can be distinguished that underlie the change (increase) in the membrane permeability of CMC: 1) an increase in the membrane permeability of CMC as a result of an increase in the load on the myocardium and stretching of its walls; 2) an increase in the membrane permeability of CMC as a result of myocardial ischemia and activation of proteolytic enzymes that can damage the cell membrane.

The first mechanism for the release of cardiospecific Tns was studied by M. Hessel et al. (Hessel et al., 2008). In their experimental study, the authors stimulated special glycoprotein receptors of CMC (integrins) that are sensitive to myocardial stretching. To model myocardial stretching and activation of integrins, the researchers used the RGD (Arg–Gly–Asp) tripeptide, which is a potent integrin agonist and is part of fibronectin and other regulatory proteins of the extracellular matrix (Ross and Borg, 2001). The authors particularly note that myocardial stretching is not associated with ischemic and necrotic processes in the cardiac muscle tissue, which indicates that it was the specific mechanism of myocardial wall stretching and the activation of integrins that ensured the release of cardiospecific Tns from viable CMC (Hessel et al., 2008).

The second mechanism for increasing membrane permeability is associated with membrane damage during ischemia of CMC. As already described above, myocardial ischemia initiates changes in the metabolism of CMC and acidification (acidosis) of the intracellular space of CMC, which, in turn, will lead to the activation of proteolytic and proapoptotic enzymes. These enzymes have many targets and in addition to the specific action (fragmentation of cardiospecific Tns), they can obviously catalyze the proteolysis of proteins that make up cell organelles and membranes (Ross and Borg, 2001; Thatte et al., 2004; Khabbaz et al., 2008). Thus, this mechanism of cardiospecific Tns increase is closely interrelated with the above-described mechanism (cardiospecific Tns increase due to increased proteolytic degradation into small fragments). In general, increased membrane permeability and intracellular fragmentation of cardiospecific Tns can be considered as two interrelated and synergistic mechanisms underlying the release of cardiospecific Tn molecules from CMC. The degree of activity of these mechanisms is probably related to the severity of pathological processes. For example, short-term and/or reversible ischemia of CMC during exercise or in uncomplicated sepsis may be associated with a relatively small increase in the activity of intracellular proteolytic enzymes. In this regard, the degree of increase in serum levels of cardiospecific Tns will also be relatively small and dependent only on the cytoplasmic fraction of cardiospecific Tns (their fragmentation into small molecular fragments) and reversible membrane damage/increased membrane permeability. In pathological conditions that cause irreversible ischemia of CMC (for example, MI or severe/complicated sepsis), serum cardiospecific Tn levels increase much more significantly and the main contribution to total serum levels of cardiospecific Tns will be made by the structural fraction of cardiospecific Tns. Both the proteins of the Tn-Tpm complex and the proteins of the membranes of CMC will be more actively fragmented (cleaved) and therefore the degree of release of cardiospecific Tns in these pathologies will be higher. The further prognosis of patients suffering from both cardiac and non-cardiac pathologies is also associated with the degree of increase in serum levels of cardiospecific Tns, which indicates the depth and nature of damage to cardiac muscle tissue.

## Release of Cardiospecific Tns as a Result of Small-Scale (Subclinical) Necrosis of CMC

A possible mechanism underlying the release of cardiospecific Tns is small-scale necrotic processes, which can be caused by both ischemia and inflammatory-toxic processes, imbalances in the neurohumoral system.

So, according to some researchers, regular heavy physical exertion, myocardites and stressful situations can cause subclinical damage to myocardial tissue (death of single CMC), which can subsequently be associated with the formation of relatively small areas of fibrosis and an increased risk of sudden cardiac death (Graham et al., 2004). So, for example, the adverse effect of serious and/or intense physical activity is confirmed by a number of studies and described clinical cases in which sudden cardiac death was recorded in athletes (Wasfy et al., 2016; DeFroda et al., 2019; Aune et al., 2020; Sollazzo et al., 2021).

Some studies registered extremely high levels of cardiac markers, including cardiospecific Tns in the blood serum of athletes after serious and prolonged physical activity (Klinkenberg et al., 2016a; Marshall et al., 2020; Martínez-Navarro et al., 2020), which is also a reason for discussing possible small-scale necrotic processes. A contradictory argument is a clinical study using magnetic resonance imaging with gadolinium (contrast) that revealed no signs of necrosis and sclerosis in the cardiac muscle tissue of athletes (O'Hanlon et al., 2010). However, the limitation of this method is its relatively lower sensitivity compared to laboratory biomarkers of myocardial necrosis and fibrosis. In addition, the authors examined only 17 athletes (average age = 33.5 ± 6.5 years) who were in good physical condition and well prepared for exercise.

Although during psycho-emotional stress the level of cardiospecific Tns increase is relatively small (rarely exceeds the levels of the 99th percentile in the isolated effect of stress), it cannot be considered a safe process (Eggers, 2013; Lazzarino et al., 2013). The constant influence of stress is considered as a risk of developing CVD and may be one of the triggers of MI (Yamaji et al., 2009; Iwaszczuk et al., 2021). A number of molecules released during stress (for example, cortisol, catecholamines) increase myocardial oxygen demand, thereby contributing to the development of relative ischemia of CMC.

## Release of Cardiospecific Tns From Non-Cardiac Cells

One of the controversial but hypothetically possible mechanisms underlying the increase in serum levels of cardiospecific Tns is the release of these molecules from non-cardiac cells. Several experimental and clinical studies indicate the expression of cardiospecific Tn molecules in skeletal muscle cells (Ricchiuti and Apple, 1999; Messner et al., 2000; Bakay et al., 2002) and the walls of large vessels (Rusakov et al., 2015a; Rusakov et al., 2015b), which allows us to consider these organs as possible sources of serum levels of cardiospecific Tns. Thus, American biochemists (V. Ricchiuti and F. Apple), using polymerase chain reaction (PCR), revealed the expression of messenger RNA of cardiospecific TnT in the skeletal muscle tissue of adults suffering from end-stage CKD and hereditary skeletal myopathy (Duchenne muscular dystrophy). Cardiospecific TnI messenger RNA was not detected in skeletal muscles of patients suffering from these pathologies and in skeletal muscles of healthy people. In addition, no signs of cardiospecific TnT expression were detected in the skeletal muscles of healthy people (Ricchiuti and Apple, 1999), which indicates the possible expression of one type of cardiospecific Tns (T) only in the presence of the indicated pathologies. In another study, B. Messner et al. confirmed the possibility of extracardiac expression of cardiospecific TnT in patients with skeletal myopathies. The researchers, using PCR, found messenger RNA of cardiospecific TnT in patients with primary sarcoglycanopathy and Duchenne muscular dystrophy (Messner et al., 2000). In some patients with skeletal myopathies, in addition to cardiospecific TnT messenger RNA, the expression of cardiospecific TnI messenger RNA was observed (Messner et al., 2000). However, in these studies, the authors did not measure serum levels of cardiospecific Tns in patients with myopathies and CKD. This is an important limitation of these studies because it does not answer the question: can the expression of cardiospecific Tns in skeletal muscles lead to an increase in serum levels of cardiospecific Tns in patients with CKD or hereditary skeletal myopathies? In addition, there should be mentioned several other studies, the results of which contradict the above data on non-cardiac expression (Bodor et al., 1995; Hammerer-Lercher et al., 2001; Schmid et al., 2018). For example, G. Bodor et al. conducted a study and concluded that cardiospecific Tns are not expressed in skeletal muscle tissue in patients with Duchenne muscular dystrophy and polymyosites (Bodor et al., 1995). Other research groups led by A. Hammerer-Lercher and J. Schmid also did not find signs of expression of cardiospecific Tns in skeletal muscles (Hammerer-Lercher et al., 2001; Schmid et al., 2018).

A second potential non-cardiac source of cardiospecific Tns release is the walls of large veins (venae cavae and pulmonary veins). Some studies report only the presence of expression of cardiospecific Tns in the walls of these veins, but do not describe the possible role of these troponins in diagnostics (Rusakov et al., 2015a; Rusakov et al., 2015b). Hypothetically, it can be believed that damage or stretching of the walls of these large veins can lead to the release of cardiospecific Tn molecules into the bloodstream.

Thus, due to the fact that data on extracardiac expression are either insufficient or contradictory, further research is needed to validate this mechanism.

The mechanisms of cardiospecific Tns release described above and their diagnostic value are summarized in Table 3.

**TABLE 3:** Release of cardiospecific Tns from CMC: mechanisms and diagnostic role.

Mechanism	Diagnostic value	Sources
CMC cell necrosis	This is the main proven mechanism underlying the increase in cardiospecific Tns in MI. CMC necrosis will result in the release of all molecules (biomarkers) from the cell into the bloodstream	Thygesen et al. (2012), Thygesen et al. (2018), Eckner et al. (2020)
Release of cardiospecific Tns as a result of the processes of regeneration and renewal of CMC	The renewal of CMC gradually occurring throughout life, hypothetically, may be associated with normal (less than the upper limit of the 99th percentile) concentrations of cardiospecific Tns in the bloodstream	Bergmann et al. (2009), White (2011), Bergmann et al. (2012)
Release of cardiospecific Tns as a result of apoptosis of CMC	It has been proven that apoptosis of CMC (without signs of necrosis) is accompanied by an increase in the serum concentration of cardiospecific Tns. Thus, any physiological (physical activity, old age) and pathological (HF, AH, COPD, etc.) conditions that enhance apoptosis may be accompanied by the release of cardiospecific Tns from CMC and an increase in serum levels	Cheng et al. (1995), Park et al. (2017), Felker and Fudim (2018), Weil et al. (2018), Gherasim (2019), Aengevaeren et al. (2021)
Release of cardiospecific Tns as a result of the formation of membrane vesicles on the surface of CMC	Membrane vesicles (blebbing vesicles) formed on the surface of the plasma membrane of CMC, hypothetically, may contain cytoplasmic proteins, including cardiospecific Tns. The number of membrane vesicles increases during ischemia of CMC and may be associated with the release of cardiospecific Tns into the bloodstream	Piper et al. (1984), Schwartz et al. (1984), Siegmund et al. (1990)
Intracellular proteolytic degradation of cardiospecific Tn molecules into small cardiospecific Tn fragments and the release of the latter through the intact membrane of CMC	Molecules of cardiospecific Tns can be fragmented/destroyed by the action of certain proteolytic enzymes: calpain, thrombin, matrix metalloproteinases. As a result of the action of these enzymes, there can form small fragments of cardiospecific Tn molecules, which, due to their size, have a higher probability of release from the cell. This mechanism may have high clinical significance: for example, all those physiological and pathological conditions and/or drugs that affect the activity of these proteolytic enzymes can also affect the release of cardiospecific Tns and their concentration in the bloodstream	Feng et al. (2001), Qun Gao et al. (2003), Lin et al. (2013), Streng et al. (2016), Katrukha et al. (2017), Parente et al. (2021)
Release of cardiospecific Tns as a result of increased membrane permeability of CMC	An increase in the release of cardiospecific Tn molecules into the bloodstream is observed in case of an increase in the membrane permeability of CMC, which is characteristic of myocardial ischemia, an increase in preload and stretching of the heart wall	Ross and Borg (2001), Thatte et al. (2004), Hessel et al. (2008), Khabbaz et al. (2008)
Release of cardiospecific Tns as a result of small-scale (subclinical) necrosis of CMC	The death of a small number of CMC may not manifest itself clinically and instrumentally (since these are relatively low-sensitivity methods), but HS methods of detection can register such subclinical lesions. Possible causes of subclinical necrosis of CMC are ischemia, inflammatory-toxic processes and imbalances in the neuroendocrine system	Martínez-Navarro et al. (2020), Marshall et al. (2020), O'Hanlon et al. (2010), Lazzarino et al. (2013)
Release of cardiospecific Tns from non-cardiac cells	This is a controversial mechanism of increased levels of cardiospecific Tns in the bloodstream. In the literature, there are works confirming the expression of cardiospecific Tns in skeletal muscle tissue in patients with CKD and hereditary skeletal myopathies, as well as studies that refute this hypothesis	Bodor et al. (1995), Messner et al. (2000), Hammerer-Lercher et al. (2001), Bakay et al. (2002), Rusakov et al. (2015a), Rusakov et al. (2015b), Schmid et al. (2018)

## Circulation of Cardiospecific Tns in Blood Plasma: Influencing Factors and Diagnostic Role

The second major stage of the metabolic pathway of cardiospecific Tns is circulation in the bloodstream. At this stage, the molecules of cardiospecific Tns are influenced by a number of factors (activity of proteolytic enzymes, kinases, phosphatases, the state of kidney function and the reticuloendothelial system, etc.), which can affect serum levels of cardiospecific Tns and, therefore, their diagnostic value. The molecules of cardiospecific Tns released into the bloodstream are represented by a heterogeneous fraction (a significant variety of different forms of troponin molecules): free troponins; combined complexes consisting of several free forms of cardiospecific Tns (for example, cardiospecific TnI + TnC, cardiospecific TnI + cardiospecific TnT, etc.) and small fragments of cardiospecific Tns (Anderson et al., 1995; Maekawa et al., 2003; Bates et al., 2010; Streng et al., 2016; Katrukha et al., 2017; Zahran et al., 2018; Vylegzhanina et al., 2019). All of the above forms of troponin molecules can undergo oxidation, glycosylation, phosphorylation and dephosphorylation processes, which leads to the formation of very diverse forms (varieties) of troponin proteins. Modifications of troponin proteins can affect such an important parameter as the half-life (half-decay) of cardiospecific Tns. This parameter has not only fundamental, but also high practical importance, since with an intensification of the breakdown of troponin proteins, their concentration and the “diagnostic window” can decrease, and with a weakening of the breakdown of cardiospecific Tns, their serum levels and the duration of the diagnostic window can increase. The researchers estimate that the half-life of cardiospecific TnT in the bloodstream is approximately 2 h, however, for many other forms, the half-life is controversial and unknown (Katus et al., 1989; Labugger et al., 2000; Gaze and Collinson, 2008). The cardiospecific TnI molecule is much less stable in the bloodstream, since it actively undergoes the processes of oxidation, phosphorylation and fragmentation (Bodor et al., 1997; Katrukha et al., 1997). The latter, in turn, as noted above for cardiospecific TnT, depend on the activity of these enzymes, the presence of concomitant pathologies that can affect the activity of these enzymes, the intake of drugs that affect the catalytic activity of proteolytic enzymes and the functional state of the organs responsible for the elimination of molecules of cardiospecific Tns. For example, increasing the activity of the enzyme thrombin (which has been shown to cause specific fragmentation of cardiospecific TnT) (Katrukha et al., 2017) can reduce the half-life of cardiospecific TnT and its concentration in the bloodstream. It is logical that taking drugs that reduce thrombin activity (for example, direct thrombin inhibitors, direct and indirect anticoagulants) can increase the half-life of cardiospecific TnT and the duration of the diagnostic window. As examples of the influence of other factors on the duration of the circulation of cardiospecific Tns in the bloodstream, there can be named the functional state of the kidneys and the reticuloendothelial system. Thus, protein molecules of cardiac markers, including cardiospecific TnT and I, can be captured by the reticuloendothelial system (macrophages) and are destroyed there (Hayashi and Notkins, 1994; Prabhudas et al., 2014; Klichowska-Palonka et al., 2015). Based on this, the following point of view comes out: an increase in the activity of the reticuloendothelial system (for example, with hypersplenism and splenomegaly) may be accompanied by an increase in the cleavage of cardiospecific Tns and a decrease in the half-life; and a decrease in the activity of the reticuloendothelial system, on the contrary, will lead to a weakening of the cleavage of cardiospecific Tns and an increase in the half-life. Kidney function is also significantly associated with cardiospecific Tn levels and increased blood protease activity. Thus, an increase in the activity of proteolytic enzymes can lead to the formation of a large number of small fragments of troponin molecules, which, like many low molecular weight proteins, can be filtered through the three-layer glomerular (filtration) barrier of nephrons. However, the filtration rate can change both under physiological and pathological conditions, which can have a significant effect on the rate of removal of Tn fragments. With pronounced drops in filtration rate (for example, with CKD or a decrease in blood pressure), the molecules of cardiospecific Tns will not be filtered (removed) from the bloodstream into the urine, but will accumulate in the blood, which will lead to an increase in the half-life of cardiospecific Tns and prolongation of the diagnostic window (De Zoysa, 2004; Dubin et al., 2013; Di Lullo et al., 2015).

Clear evidence that cardiospecific Tn molecules can pass (filter) through the glomerular filter has been presented in several recent clinical studies due to the use of HS troponin immunoassays (Klichowska-Palonka et al., 2015; Pervan et al., 2017; Chen et al., 2020). A similar transport mechanism is probably characteristic of the filtration of cardiospecific Tns into the oral fluid through the blood-salivary barrier, which is also supported by several pilot studies that have established a correlation between serum and salivary troponin levels (Mirzaii-Dizgah and Riahi, 2013a; Mishra et al., 2018; Chaulin et al., 2019; Chaulin et al., 2020c).

The search for specific mechanisms of proteolytic cleavage of cardiospecific Tns in the bloodstream is of great practical importance, since it will optimize laboratory diagnostics: in particular, there is a possibility of developing antibodies directed against individual fragments of cardiospecific Tns or introducing inhibitors of the main proteolytic enzymes that catalyze troponin proteolysis into diagnostic test systems to reduce interference and more thoroughly interpret the test results, taking into account comorbidities that affect the activity of enzymes breaking down cardiospecific Tns, etc. Unfortunately, the number of fundamental studies devoted to the investigation of the processes of proteolytic cleavage of cardiospecific Tns in the bloodstream is extremely small. And to date, only one specific mechanism is known, described in the study by Katrukha et al. (2017), Katrukha et al. (2018). According to the results of this study, the enzyme thrombin catalyzes the cleavage of the full-length molecule of cardiospecific TnT (the molecular weight is 35 kDa) in the region of the peptide bond between amino acids 68 and 69 into two fragments, one of which is larger (the molecular weight is 29 kDa), and the second—smaller (the molecular weight is 6 kDa) (Katrukha et al., 2017). As noted above, any significant effect on thrombin activity (for instance, the use of anticoagulants) can influence the fragmentation of cardiospecific TnT and, accordingly, its diagnostic value.

In general, a number of research groups studying the processes of proteolytic cleavage in the bloodstream report the presence of a very large number of fragments (approximately several tens) of cardiospecific Tn molecules, which have different sizes (molecular weights from several kDa to 30 or more kDa), stability and half-life in the bloodstream (from several hours to a day) and conditions of formation (physiological conditions, the degree of severity and progression of ischemia, reperfusion time, etc.) (Anderson et al., 1995; Maekawa et al., 2003; Bates et al., 2010; Streng et al., 2016; Katrukha et al., 2017; Zahran et al., 2018; Vylegzhanina et al., 2019). A. Vylegzhanina et al. studied the composition of troponin complexes in patients with MI (Vylegzhanina et al., 2019). The researchers have identified the following main forms of cardiospecific Tns in MI: a ternary complex consisting of full-size cardiospecific TnT and I and TnC; a ternary complex consisting of truncated cardiospecific TnI and integral cardiospecific TnT and TnC; a binary complex consisting of truncated cardiospecific TnI and TnC, as well as a number of short fragments of cardiospecific TnT and cardiospecific TnI, formed mainly from the central part of the molecules. As MI progressed, there was a decrease in the number of ternary complexes consisting of full-size cardiospecific Tns and an increase in the number of ternary and binary complexes consisting of truncated troponins, as well as an increase in the level of fragments of cardiospecific Tns (Vylegzhanina et al., 2019). Such changes in the heterogeneous fraction of cardiospecific Tns are most likely due to an increase in the activity of proteolytic enzymes, which increase with the progression of ischemia and MI, and, accordingly, cause fragmentation (truncation) of troponin proteins.

Very interesting data are presented by researchers S. Zahran et al. This research group studied the degree of proteolytic degradation of cardiospecific TnI in patients with varying degrees of ischemia and damage to cardiac muscle tissue (Zahran et al., 2018). The researchers noted that the degree of proteolytic cleavage of cardiospecific TnI increases with an increase in the severity of ischemia and myocardial injury: the highest degree of proteolytic cleavage of cardiospecific TnI was characteristic of patients with ST-segment elevation MI, while in patients with non-ST-segment elevation MI the degree of cardiospecific TnI degradation was significantly lower. The authors also found a decrease in the degree of proteolytic degradation of cardiospecific TnI after reperfusion, which can probably be used to assess the quality of reperfusion. It is quite remarkable that the degree of proteolytic degradation of cardiospecific TnI had a higher diagnostic value in MI than the total serum concentration of cardiospecific TnI (Zahran et al., 2018).

Summing up the role of the stage of cardiospecific Tns circulation, we should once again emphasize its potentially high diagnostic role for practical medicine. At the moment, this stage is a relatively poorly studied area of the biology of cardiospecific Tns. The main directions of further work in this area to increase the diagnostic role of cardiospecific Tns should be:

1) Study of the fundamental specific mechanisms of proteolytic degradation of cardiospecific Tns in the bloodstream both under normal conditions and under the conditions of simulated concomitant pathologies. This requires a targeted and thorough study of the potential effect of individual serum proteolytic enzymes (for example, specific thrombin-mediated degradation of cardiospecific TnT) (Brian Gibler et al., 1987).
2) Search for specific fragments of cardiospecific Tns, which are released at the earliest possible time after the onset of myocardial ischemia and the creation of antibodies to them, which will increase the sensitivity and specificity of troponin immunoassays.
3) Search for specific fragments of cardiospecific Tns, which have a small molecular weight and are able to pass through the glomerular and blood-salivary barriers. The creation of antibodies to these fragments will make it possible to develop specific HS test systems for the analysis of non-invasive biological fluids (urine and oral fluid) and for the introduction of new methods of non-invasive diagnostics and monitoring of CVD, including MI, into routine clinical practice.
4) Study and identification of potentially possible specific mechanisms of proteolytic cleavage of cardiospecific Tns under the action of other (non-ischemic) factors. This will allow the development of specific troponin immunoassays to identify those fragments that, for example, will increase exclusively with stretching of the myocardium or exclusively with an increase in the activity of the adrenergic nervous system and an increase in β-AR stimulation, etc. Thus, it will be possible to carry out a more specific diagnosis of non-ischemic myocardial damage in some physiological and pathological conditions not associated with ischemia of the cardiac muscle tissue.

## Removal of Cardiospecific TnI From the Bloodstream: Mechanisms and Diagnostic Role

The final stage of the metabolic pathway of cardiospecific Tns in the bloodstream is as important as the other two stages (release and circulation). Both of these stages are closely related to the terminal stage of the metabolic pathway of cardiospecific Tns. So, for example, when small fragments are released from CMC (as a result of intracellular proteolytic cleavage of cardiospecific Tns), they will obviously be almost immediately removed from the bloodstream by filtration through the glomerular and blood-salivary barriers. When larger fragments of cardiospecific Tns and/or binary and ternary complexes are released, filtration of these molecules is unlikely due to their large size (molecular weight).

The circulation of cardiospecific Tns is equally closely related to the removal of these molecules. So, for example, with a higher activity of serum proteolytic enzymes, the process of degradation of cardiospecific Tn molecules will be more active, which will lead to more rapid formation of small cardiospecific Tn molecules and their filtration (removal) from the bloodstream.

In general, today, the mechanism of filtering cardiospecific Tns through the glomerular barrier is one of the main and definitively proven ways to remove cardiospecific Tns from the bloodstream. The inferential (indirect) evidence is that when the filtration rate decreases (for example, in CKD), cardiospecific Tn molecules accumulate in the bloodstream and their serum levels begin to rise sharply in those patients who do not have any signs of CVD and damage of CMC. And the more the kidney function is suppressed (i.e., the lower the filtration rate is), the higher the concentration of cardiospecific Tns in the bloodstream rises (Dubin et al., 2013; Di Lullo et al., 2015; Han et al., 2020). In other pathological conditions that are accompanied by inhibition of the filtration rate, for example, in sepsis, serum levels of cardiospecific Tns are positively correlated with serum creatinine levels (Røsjø et al., 2011; Wilhelm et al., 2014), which also accumulate due to a decrease in the filtration capacity of nephrons. From a pathogenetic point of view, any conditions accompanied by a drop in the filtration rate can contribute to the accumulation of troponins. This fact, of course, should be taken into account by medical practitioners when interpreting the results.

Recent clinical studies by several research groups can be considered as valuable evidence of the existence of a mechanism for the elimination of cardiospecific Tns across the filtration barrier (Pervan et al., 2017; Chen et al., 2020). A key feature of these studies is the use of HS troponin immunoassays, which can detect small concentrations (from several ng/L to several tens of ng/L) of cardiospecific Tns in urine. According to these studies, it is also noteworthy that there is a possibility of non-invasive assessment of myocardial damage in AH and diabetes mellitus (Pervan et al., 2017; Chen et al., 2020), which is very convenient for non-hospital and outpatient settings. This will allow monitoring the patient’s condition, assessing the prognosis, and, on its basis, choosing/correcting the tactics of further management of patients, including their treatment. However, it should be noted that these methods have not yet been completely validated and research work in this direction should be continued before introducing new non-invasive methods for diagnosing and monitoring CVD in routine clinical practice.

One of the key and very labile factors affecting the glomerular filtration rate (including the rate of removal of cardiospecific Tns from the bloodstream) is blood pressure. So, with a decrease in blood pressure, the filtration rate will slow down and the degree of removal of cardiospecific Tns from the bloodstream will decrease. This mechanism, in particular, can contribute to the fact that the molecules of cardiospecific Tns will increase much higher and circulate in the bloodstream for longer in pathological conditions accompanied by a sharp drop in blood pressure. This can be typical for large-focal myocardial infarctions, which are often accompanied by a sharp decrease in blood pressure (cardiogenic shock), and the degree of increase/duration of circulation of cardiospecific Tns in the bloodstream can be considered as a prognostically unfavorable sign (Daly et al., 2020). With an increase in blood pressure, the filtration rate may increase, and more cardiospecific Tn molecules will be filtered from the bloodstream into the urine. The evidence for a possible role of this mechanism comes from a clinical study showing that urinary troponin levels are higher in hypertensive patients than in those with normal blood pressure or those taking antihypertensive drugs (Pervan et al., 2017).

Another way of cardiospecific Tns removal is associated with the activity of the reticuloendothelial system, the cells of which capture protein molecules of cardiac markers from the bloodstream and cause their intracellular proteolytic cleavage (Hayashi and Notkins, 1994; De Zoysa, 2004; Fridén et al., 2017; Muslimovic et al., 2020). The clinical significance of this mechanism for removing cardiospecific Tns (as opposed to the mechanism for removing cardiospecific Tns through the glomerular filter) is difficult to judge, since there are no similar well-controlled clinical studies confirming the possibility of a significant increase in serum levels of cardiospecific Tns in case of the reticuloendothelial system dysfunction. In addition, in contrast to CKD, dysfunctions of the components of the reticuloendothelial system are much less common.

**FIGURE 3:**
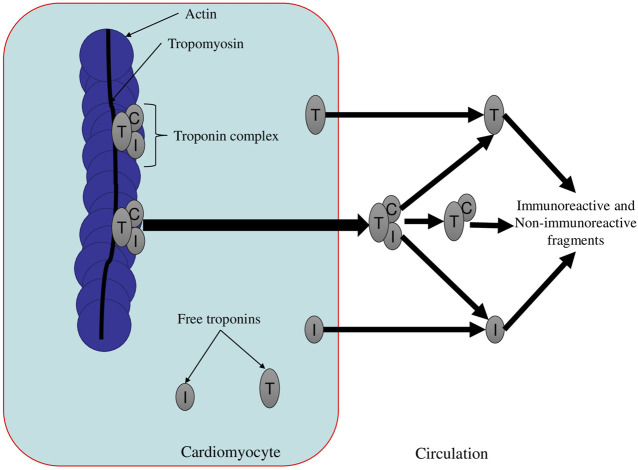
The main forms of circulating cardiospecific troponins (Gaze and Collinson, 2008). Description. The troponin complex is released from damaged cardiomyocytes (for example, in necrosis) in various molecular forms. The triple complex (TnT-TnI-TnC) degrades into a binary complex and free cardiospecific Tns. Then the binary complex also degrades into free Tns and troponin fragments (both immunoreactive and non-immunoreactive fragments). Free cardiospecific Tns can be released from cardiomyocytes with minor injuries (psychoemotional stress and physical exertion) and then also degrade into free cardiospecific troponins (TnT and TnI) and their fragments (Gaze and Collinson, 2008).

As noted earlier, the proteolytic cleavage of troponin molecules in the bloodstream is an extremely understudied mechanism for the elimination of cardiospecific Tns. As a result of this mechanism, a large number of small fragments of cardiospecific Tns are formed, which can be immunoreactive (can interact with antibodies and be detected by immunoassays) and non-immunoreactive fragments (which will not interact with antibodies) (Figure 3) (Wu and Morrow, 2006). From the point of view of laboratory diagnostics, non-immunoreactive fragments of troponins can be considered already removed from the bloodstream, since they will not bind to antibodies and thus will not have any effect on the result of laboratory diagnostics of MI or any other pathology. To elucidate the mechanism of cardiospecific Tn removal by proteases, well-controlled basic research is needed to thoroughly investigate the role of individual serum proteolytic enzymes in the degradation of cardiospecific Tn molecules in the bloodstream.

Thus, there can be distinguished 3 main mechanisms of elimination of cardiospecific Tns from the bloodstream: 1) elimination (filtration) of cardiospecific Tns through the glomerular barrier, 2) removal of cardiospecific Tns from the bloodstream by cells of the reticuloendothelial system, 3) proteolytic cleavage of cardiospecific Tn molecules and/or their fragments in the bloodstream to non-immunoreactive forms. Taking into account the analysis of the literature, the main mechanism for the removal of cardiospecific Tns, in my opinion, is the elimination of Tns through a three-layer filtration (glomerular) barrier. This mechanism can have a significant impact on the diagnostics of CVD, including MI, since impaired removal of cardiospecific Tns from the bloodstream is often accompanied by a significant increase in serum levels of cardiospecific Tns. In addition, many patients have comorbid pathologies, among which kidney damage (CKD) is relatively common. Many other common diseases, for example, diabetes mellitus, sepsis, are also often complicated by CKD. Thus, they may increase serum levels of cardiospecific Tns in patients having no signs of CVD.

Removal of cardiospecific Tns from the bloodstream by glomerular filtration may have an important impact on rapid algorithms for diagnostics/exclusion of MI. Thus, the research group led by P. Kavsak reported that the currently established upper threshold levels of troponins (99th percentile) for the diagnostics/exclusion of MI can be used only for patients with an optimal glomerular filtration rate (≥90 ml/min) (Kavsak et al., 2018). In patients who have lower glomerular filtration rate values, cardiospecific Tns levels will increase due to impaired elimination, which can lead to overdiagnosis of MI if medical practitioners do not take kidney function (filtration rate value) into account. Thus, it is necessary to stratify the threshold values of cardiospecific Tns taking into account different values of the filtration rate and, in particular, to develop special algorithms for diagnostics/exclusion of MI for patients who suffer from concomitant CKD.

Finally, the filtration of cardiospecific Tn fragments through the blood-brain barrier into the cerebrospinal fluid and through the blood-salivary barrier into saliva can be considered as additional potential mechanisms for the removal of cardiospecific Tns. As evidence of the existence of these mechanisms, one can consider studies (Panteghini et al., 2008; Tate et al., 2010; Mirzaii-Dizgah and Riahi, 2013a; Mirzaii-Dizgah and Riahi, 2013b; Streng et al., 2013; Gore et al., 2014; Klinkenberg et al., 2014; Jarolim, 2015; Klichowska-Palonka et al., 2015; Abdul Rehman et al., 2017; Fournier et al., 2017; Pervan et al., 2017; Boeddinghaus et al., 2018; Garcia-Osuna et al., 2018; Katrukha et al., 2018; Monneret et al., 2018; Vogiatzis, 2018; Wildi et al., 2018; Bohn et al., 2019; Chaulin et al., 2019; Rocco et al., 2019; Romiti et al., 2019; Chaulin et al., 2020c; Chen et al., 2020; Chaulin et al., 2020d; Giannitsis et al., 2020; Zaninotto et al., 2020; Bahbah et al., 2021; Chaulin and Duplyakov, 2021b), which reported on the detection of cardiospecific Tns in the cerebrospinal fluid and saliva. The investigation of cardiospecific Tns in the cerebrospinal fluid can be used in forensic medicine, and the investigation of cardiospecific Tns in saliva—in clinical practice for diagnostics and monitoring of CVD, including MI. Overall, more research is needed to validate these diagnostic capabilities.

## Circadian Rhythms of Cardiospecific Tns: Possible Mechanisms of Formation and Diagnostic Role

The activity of many systems (organs, tissues, and cells) of our body changes cyclically during the day (with the change of day and night), which is commonly called circadian or diurnal rhythms. Circadian rhythms are an evolutionarily developed mechanism necessary to maintain optimal functioning of the body and adapt to changing environmental conditions (Patke et al., 2020; Chaulin and Duplyakov, 2021e; Chaulin, 2021f; Chaulin, 2021g).

Due to the fact that the tissues and cells of our body change, there is a change in the concentration of a number of molecules (for example, hormones, metabolic products), which are produced or metabolized by these tissues and cells. Many of these molecules are laboratory biomarkers, the concentration of which is used to diagnose diseases (Cribbet et al., 2016; Thosar et al., 2018). This must be taken into account in routine clinical practice, since changes in the concentration of biomarkers caused by natural circadian rhythms can be mistakenly interpreted as diagnostic signs and, accordingly, lead to diagnostic errors. Certain hormones, the release of which varies from day to night, can affect a number of other laboratory parameters that must also be considered when interpreting laboratory diagnostic results.

Recent clinical studies have reported that bloodstream levels of cardiospecific Tns are dependent on circadian rhythms. These studies used HS troponin immunoassays able to detect small fluctuations in the concentration of cardiospecific Tns in the bloodstream (at the level of several ng/L) (Klinkenberg et al., 2016b; van der Linden et al., 2016). For example, L. Klinkenberg et al. revealed changes in cardiospecific TnT concentration (detected by a HS method) in patients without signs of CVD. At the same time, the maximum levels of troponins were recorded in the morning (16.2 ng/L at 8:30), and the minimum—in the evening (12.1 ng/L at 19:30). In addition, when analyzing the hourly curve of serum levels of cardiospecific Tns, the researchers found very regular and gradual changes: for example, from the maximum morning concentrations of cardiospecific Tns there was a gradual decrease to the evening (minimum) concentrations of cardiospecific Tns, and then there was a gradual increase in concentrations to the maximum morning values (Klinkenberg et al., 2016b). However, the researchers noted that such relatively minor circadian fluctuations in Tns would not have a significant impact on diagnostic algorithms for MI, but should be considered for screening purposes. The levels of cardiospecific TnI (also detected by a HS immunoassay) changed over a 24-h period by no more than 1 ng/L, i.e., had no significant circadian rhythms (Klinkenberg et al., 2016b). However, this study investigated the circadian rhythms of Tns only in healthy people, but in conditions of concomitant pathologies (especially with damage to those organs that affect the metabolism of cardiospecific Tns), fluctuations in the circadian rhythms of cardiospecific Tns can be much more pronounced. Thus, in patients with concomitant CKD, cardiospecific TnT and cardiospecific TnI concentrations changed more significantly during the day. N. van der Linden et al. (van der Linden et al., 2016) reported that the maximum fluctuations in cardiospecific TnT concentration in a patient with CKD during the 24-h investigation period were about 50 ng/L, while the fluctuations in cardiospecific TnT levels during 1 h were about 20 ng/L, which, by the way, is a very significant contribution to the laboratory diagnosis of MI. So, for example, if we take into account modern algorithms for diagnostics of non-ST-segment elevation MI (Table 1) (Qin et al., 2021) (where the change in the levels of cardiospecific Tns within 1–2 h by only 5–10 ng/L is diagnostically significant), we can say that cardiospecific TnT circadian rhythms may affect the diagnostics of AMI and contribute to overdiagnosis (van der Linden et al., 2016). Cardiospecific TnI levels showed slightly higher fluctuations in concentration during the day compared to the study by L. Klinkenberg et al. (Klinkenberg et al., 2016b), however, they would not reach the thresholds of 5–10 ng/L and thus would not have a significant effect on one- and 2-h algorithms of MI diagnostics.

The precise mechanisms of the formation of circadian rhythms of cardiospecific Tns are unknown, however, it can be assumed that they will be associated with changes in the functional activity of those organs, tissues and cells that can somehow affect the metabolic pathway of cardiospecific Tns, in particular the stages of their release into the bloodstream, the stage of circulation (for example, the effect on the activity of proteolytic enzymes that cause the cleavage of troponins in the bloodstream) or the elimination stage (for example, the effect on the functional state of the kidneys). Among the most probable mechanisms for the formation of circadian rhythms of cardiospecific Tns, in my opinion, there are circadian fluctuations in the activity of the cortex and medulla of the adrenal glands, of the thyroid gland, and the activity of enzymes of the hemostatic system (Tofler et al., 1987; Panza et al., 1991; Suárez-Barrientos et al., 2011; Tsareva et al., 2019; Chaulin and Duplyakov, 2021f). A possible rationale for the formation of circadian rhythms is that peak cardiospecific Tn concentrations occur in the morning period, being the period of the maximum activity of the adrenal glands (producing elevated levels of catecholamines, cortisol), the thyroid gland (producing thyroid hormones, which can enhance the effects of catecholamines on CMC). The increased activity of these organs also coincides with their main effects on the cardiovascular system, namely, in the morning period, patients have the highest heart rate, and the blood pressure is higher than in the evening-night period. In general, the increased activity of these organs is a kind of adaptive and evolutionary developed mechanism, which is necessary to maintain the period of wakefulness. However, we should take into account the negative impact of these organs and their metabolic products (for example, catecholamines, cortisol) on CMC. The evidence of the adverse effect of cortisol on CMC is a clinical study that demonstrates that increased levels of the stress hormone (cortisol) are associated with increased levels of cardiospecific TnT (Lazzarino et al., 2013). In addition, a number of researchers associate the increased high activity of the sympathoadrenal system with a larger size of the focus of myocardial necrosis in MI, the incidence of acute CVD and an unfavorable prognosis (Manfredini et al., 2004; Arroyo Úcar et al., 2012; Fournier et al., 2014; Seneviratna et al., 2015; Roos and Holzmann, 2018; Chaulin, 2021; Chaulin and Duplyakov, 2021g; Chaulin, 2021h; Chaulin and Duplyakov, 2022; Mullova et al., 2022).

A possible explanation of the reason for the fact that the circadian rhythms of cardiospecific TnT are more significant than the circadian rhythms of cardiospecific TnI consists in their biochemical features, in particular, the volume of the cytoplasmic fraction of cardiospecific TnT is almost twice the volume of the cytoplasmic fraction of cardiospecific TnI (approximately 7–8% *versus* 3–4%) (Chaulin, 2021d). Thus, the cytoplasmic fraction of cardiospecific TnT is more “mobile” and can be released into the bloodstream with an increase in the effect of a number of factors on the myocardium. There is a need for further clinical studies validating the circadian rhythms of cardiospecific Tns and their effect on the diagnostics of CVD, including MI, and for fundamental research clarifying the molecular mechanisms of the formation of circadian rhythms of cardiospecific Tns.

## Conclusion

The metabolic pathway of cardiospecific Tns includes three main stages (release of Tns from CMC, circulation of cardiospecific Tns in blood plasma, removal of cardiospecific Tns from the bloodstream), each of which can have a very significant effect on serum levels of cardiospecific Tns, i.e., on their diagnostic value.

It should be noted that many new views on the metabolism and diagnostic value of cardiospecific Tns (in particular, the role of circadian rhythms, gender- and age-related characteristics of concentrations, the possibility of detecting Tns in urine and saliva, etc.) were formed as a result of an increase in the sensitivity of troponin immunoassays.

Unfortunately, today many stages of the metabolic pathway of cardiospecific Tns and factors influencing the metabolic pathway of cardiospecific Tns are extremely poorly understood and are hypothetical and/or contradictory. In particular, the specific mechanisms of the release of cardiospecific Tns from the myocardium into the bloodstream as affected by physiological conditions and characteristics (physical exertion, stress, circadian fluctuations in the activity of organs and tissues that influence the release of cardiospecific Tn molecules) and non-ischemic pathologies, which are often accompanied by an increase in the concentration of cardiospecific Tns in the bloodstream, are little known. And factors affecting the circulation of cardiospecific Tn molecules in the bloodstream, in particular enzymes involved in the metabolism (fragmentation) of cardiospecific Tn molecules, remain unknown. The mechanisms of filtration (transport) of cardiospecific Tn molecules from the bloodstream to other biological fluids are not investigated, and, accordingly, these possibilities of non-invasive diagnostics have not been validated. Thus, the study of the metabolic pathway of cardiospecific Tns and potential factors influencing it is a relatively large and poorly studied area for further research that is needed to optimize diagnostics and validate new diagnostic capabilities(Stefanovic and Loukovaara, 2005; Zhu et al., 2007; Van Mieghem et al., 2010; Yoshida et al., 2013; Blohm et al., 2019).
